# Mechanical thrombectomy for acute multivessel occlusions with duplicated middle cerebral artery: A case report

**DOI:** 10.3389/fneur.2022.1089255

**Published:** 2023-01-09

**Authors:** Hai-Ji Han, Qiang Sang, Xi-Ming Wang, Yan-Feng Wu

**Affiliations:** ^1^Department of Neurology, The Affiliated Kezhou People's Hospital of Nanjing Medical University, Kezhou, China; ^2^Department of Neurology, The Second Affiliated Hospital of Nanjing Medical University, Nanjing, China

**Keywords:** acute ischemic stroke, duplicated middle cerebral artery, mechanical thrombectomy, multivessel occlusions, large vessel occlusion

## Abstract

Acute multivessel occlusions generally have multisite clot burden with lower successful reperfusion rates, and cerebrovascular anatomical variants increase the challenge of endovascular clot retrieval. We report a case of acute anterior multivessel occlusions patient with duplicated middle cerebral artery. Combined balloon guide catheter with stent retriever and aspiration approach has gained complete revascularization and good functional outcomes at 3 months follow-up.

## Introduction

Acute ischemic stroke (AIS) with multivessel occlusions (MVOs), usual involvement of more than one territory in the ipsilateral or bilateral anterior circulation or the anterior circulation plus the posterior circulation, accounts for 10–15% of AIS admission (1, 2). Simultaneous occlusions of two or more large or medium vessels have a poorer prognosis because of the multisite clot burden and lower successful reperfusion rates associated with a higher incidence of downstream flow disruption (3, 4). Duplicated middle cerebral artery (DMCA) is a rare intracranial vascular variant, and angiography may confuse operators and lead to missed diagnoses because one branch of the DMCA is occluded while the other branch shows a normal middle cerebral artery, which requires careful screening (5). Simultaneous occlusions of the middle cerebral artery (MCA) with the anterior cerebral artery (ACA) and DMCA decrease the rates of first-pass effect and successful recanalization. The results of using the combined balloon guide catheter (BGC) with stent retriever (SR) and distal access catheter (DAC) technique for mechanical thrombectomy (MT) showed a higher rate of the first-pass and overall complete reperfusions and shorter procedure times in patients with the internal carotid artery (ICA) or the proximal MCA occlusions (6). We describe a patient presenting acute anterior MVOs with DMCA who underwent MT using a combined technique.

## Case report

A female patient in her late 70s presented to the emergency department with left-sided MCA syndrome. The patient had a history of ischemic stroke without residual deficits, persistent atrial fibrillation, and rheumatic heart disease, which successfully underwent mitral valve replacement and aortic valve replacement in 1996 and 2001, respectively. She is taking a daily warfarin dose of 3 mg to prevent thromboembolism but has not monitored the international normalized ratio (INR) for the last month. Her baseline modified Rankin score (mRS) was 0 and the well-known last time was 7 h. National Institutes of Health Stroke Scale (NIHSS) score at admission was 17 for left-sided gaze, right-sided facial palsy, right-sided upper and lower extremity motor deficits, and diminished sensation and aphasia.

**Figure 1:**
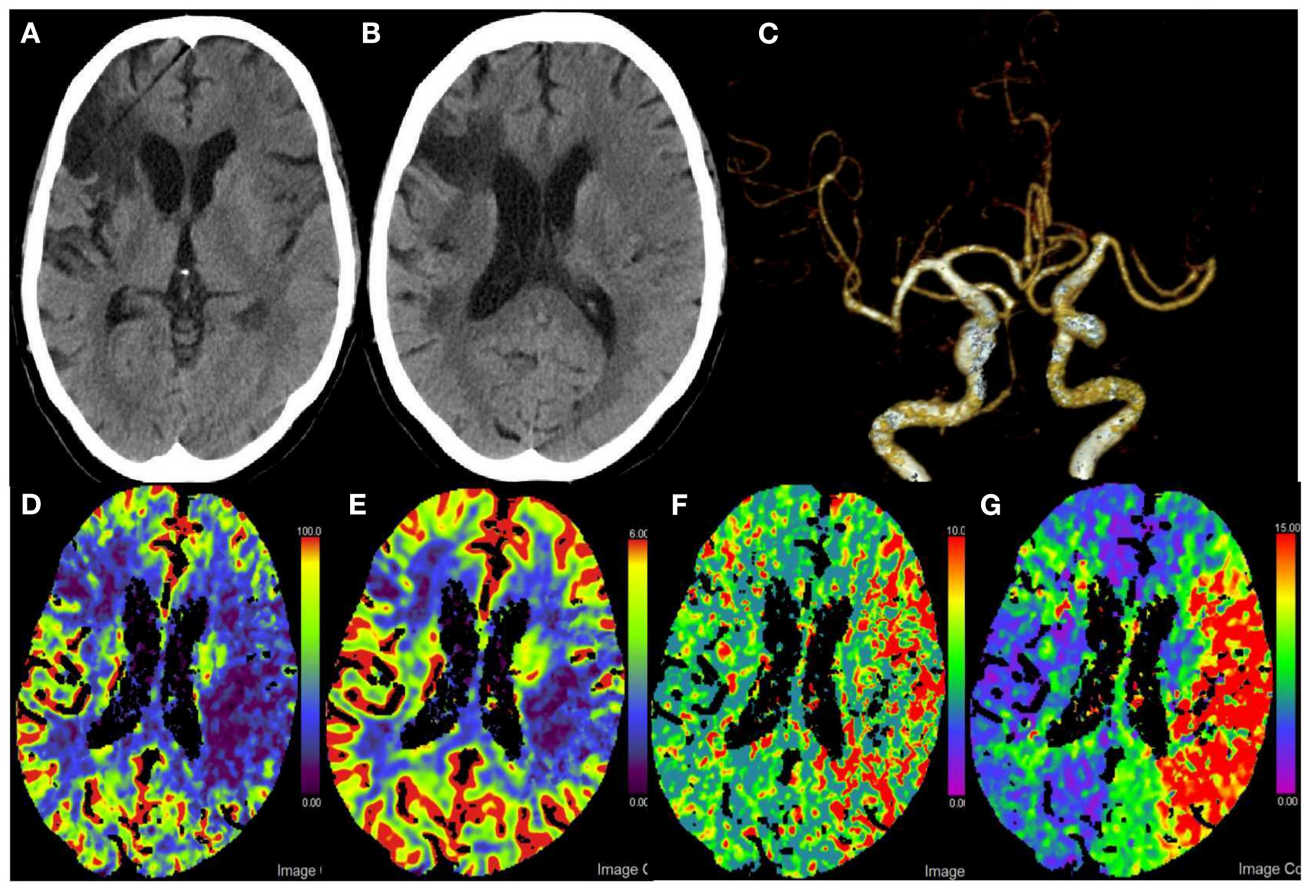
Pre-procedural imaging. **(A,B)** Axial pre-procedural CT of the head non-contrast shows the blurring of the gray-white border in the middle third and fourth of the left middle cerebral artery (MCA) territory. **(C)** Pre-procedural CT angiography demonstrating occlusion of the M1 terminus. **(D–G)** Pre-procedural CT perfusion shows decreased CBF and CBV and increased MTT and TTP in the left MCA territory. CBF suggests a large area of hypoperfusion in the left cerebral hemisphere; CBV suggests an infarct core that is smaller in extent than CBF, suggesting a mismatch in CBF/CBV, which may be good candidates for reperfusion therapy.

Non-contrast CT of the brain showed evidence of acute infarction in the left parietal lobe with an Alberta Stroke Program Early CT Score (ASPECTS) of 8 (Figures 1A, B). CT angiography (CTA) demonstrated occlusion of the left distal MCA (Figure 1C). CT perfusion (CTP) showed massive hypoperfusion with reduced cerebral blood flow (CBF) and cerebral blood volume (CBV), the left cerebral hemisphere had increased mean transit time (MTT) and time to peak (TTP), and a CBF/CBV mismatch that may be a good candidate for reperfusion therapy (Figures 1D–G). CTP data were processed using Siemens syngo *via* CT Neuroperfusion (version VB20A; Siemens Healthcare, Erlangen, Germany). Given the perfusion mismatch, the patient was subjected to mechanical thrombectomy.

**Figure 2:**
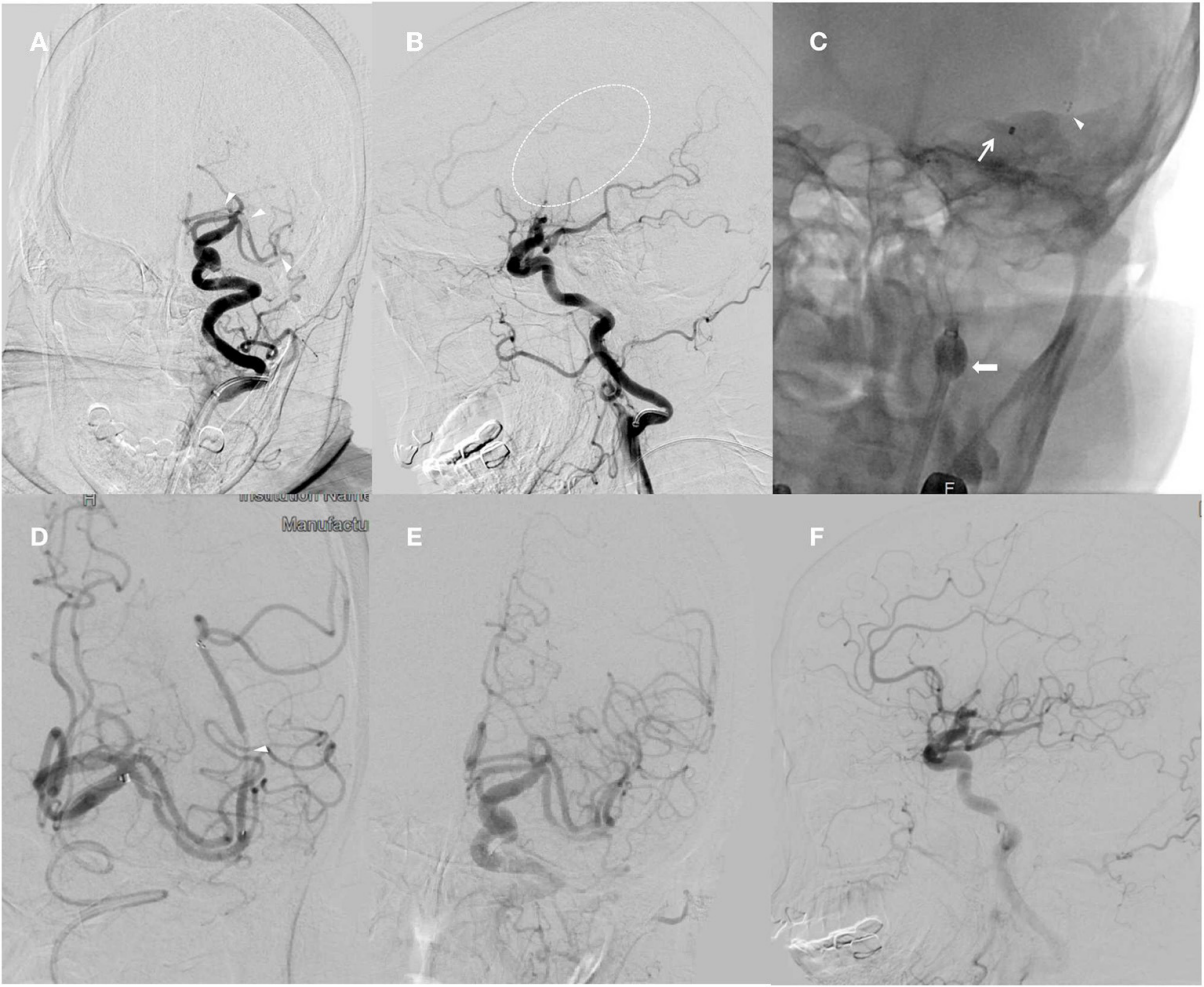
Angiographic acquisitions, left internal carotid artery injection, and anteroposterior and lateral view obtained during thrombectomy. **(A)** Demonstration of the left A1 segment of ACA, superior M1 proximal, and inferior Sylvian M2 occlusions (arrow). **(B)** Lateral view displaying abrupt cutoff of the M1 branch of the left MCA (dashed arrow). **(C)** Combined balloon guide catheter with stent retriever and distal access catheter technique used in mechanical thrombectomy procedure. The balloon of the BGC is inflated to arrest the antegrade ICA flow (thick arrow). The tip of the DAC indicates placement within the M1 segment (thin arrow). The stent retriever is placed from M1 into the terminal ICA (arrowhead). **(D)** The same procedure is repeated for the inferior Sylvian M2 occlusion (arrow). **(E, F)** Full recanalization of the MCA and ACA territory following the second pass.

Under ICA sedation, right femoral artery access was achieved within 7 h and 45 min after symptom onset using an 8-French sheath. Using a coaxial technique, a 0.087-inch SeparGate balloon guide catheter (BGC) (Ruikangtong Technology Development Co., Hunan, China) was advanced from the 5-French select catheter to the left ICA. Digital subtraction angiography (DSA) showed that the patient had MCA duplication on the left side, with proximal occlusion of the left A1 segment of ACA, superior M1 proximal, and inferior Sylvian M2 (Figures 2A, B), explaining the patient's symptoms and the perfusion deficit seen on CTP.

The BGC was positioned in the extracranial segment of the occluded ICA, and then the 6FDAC (Ruikangtong Technology Development Co., Hunan, China) was introduced, and the Rebar 18 microcatheter (ev3 Covidien, Irvine, CA, USA) was first placed on the 0.014-in Traxcess microguide wire (Microvention, Aliso Viejo, CA, USA) in the M1 segment of the occluded MCA. Based on the roadmap, a 6 × 30 mm Solitaire AB stent (ev3, Irvine, California, USA) was advanced into the occluded segment, the microcatheter was withdrawn, and the stent was deployed for 5 min. Subsequently, the stent was partially retrieved with the microcatheter and the DAC was pushed proximal to the stent. Prior to stent retrieval, the balloon of the BGC was inflated to stop the retrograde flow of the ICA (Figure 2C). The stent and microcatheter as a unit were gently pulled back into the DAC. During the aspiration process, the DAC was slowly pulled back until free blood was obtained through the aspiration syringe. After stent retraction, the balloon was immediately released to allow recirculation of the ICA. Follow-up angiography showed complete recanalization of the ACA and supra-MCA regions, consistent with a modified Thrombolysis in Cerebral Infarction (mTICI) score of 2b. The repeated treatment of the thrombus in the inferior Sylvian M2 was performed using a 4 × 20 mm Solitaire AB stent (Figure 2D), which showed complete recanalization with an mTICI score of 2b (Figures 2E, F). There was no evidence of any untoward events in either procedure.

**Figure 3:**
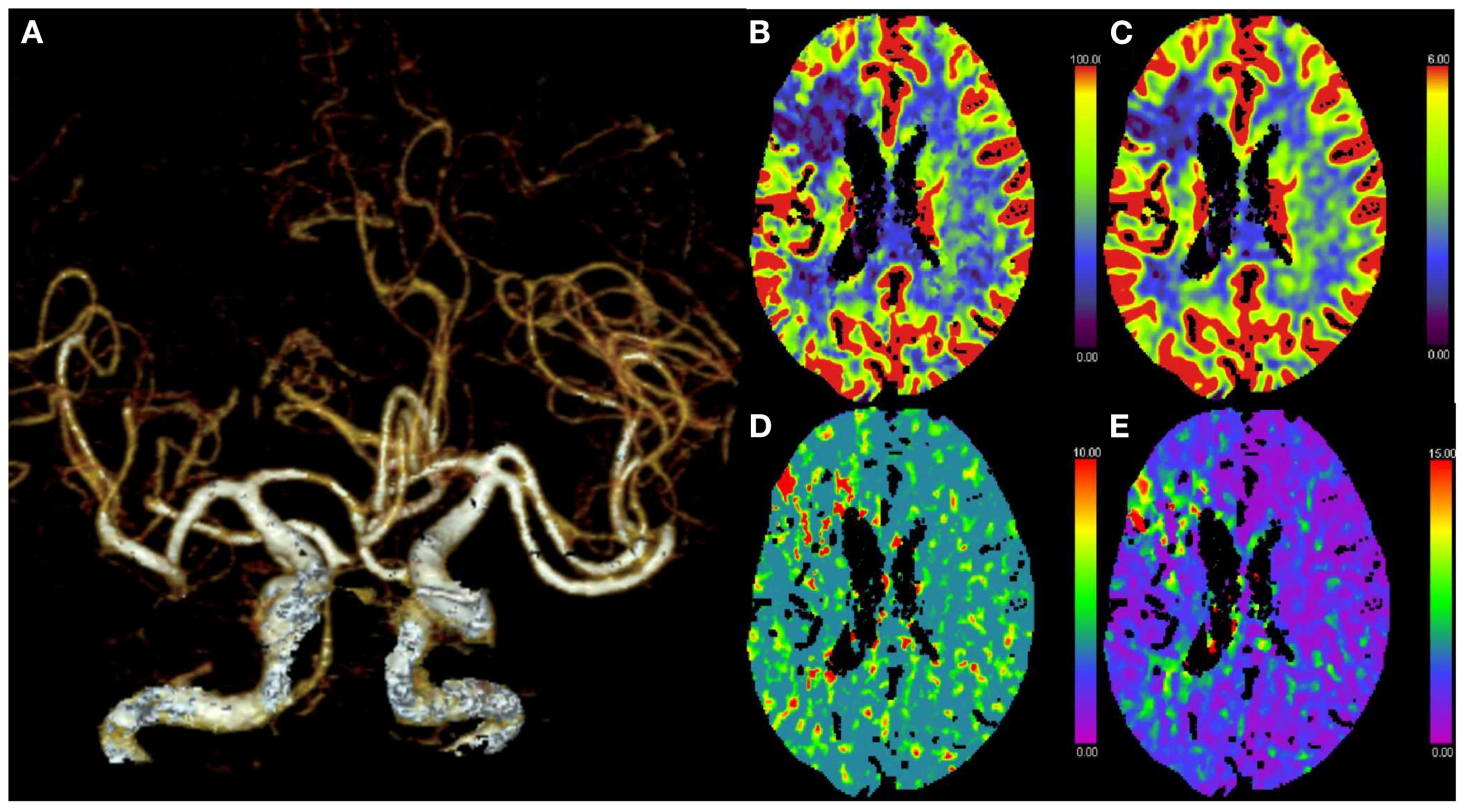
Post-treatment CT angiography (CTA) and perfusion (CTP) at 5 days. **(A)** CTA demonstrates no occlusion of the ACA and DMCA. **(B–E)** CTP shows normalization of perfusion in the left cerebral hemisphere.

Follow-up brain CT was performed immediately and 24 h after MT and demonstrated stable subacute left MCA distribution infarction with no evidence of acute hemorrhagic complications. Five days later, follow-up of CTA and CTP showed patency of ACA and DMCA complete normalization of perfusion in the left cerebral hemisphere (Figure 3). The patient was discharged 10 days after the thrombectomy with an NIHSS score of 1 for right facial palsy. At the 3-month follow-up, the mRS of the patient was 0.

## Discussion

Simultaneous MVOs are rare conditions but devastating diseases, which may be encountered during MT for AIS (7). The prevalence of MVOs is 0.34–2.0% in patients with AIS who underwent MT (2, 7). Most simultaneous MVOs are caused by cardioembolic origin or artery-to-artery embolism (2, 4). Kim et al. reported 12 patients with simultaneous ACA and MCA occlusion and found that all patients were diagnosed with atrial fibrillation (4). In our study, the patient had a history of persistent atrial fibrillation and rheumatic heart disease. Hence, it is important to evaluate cardioembolism for simultaneous MVO (4).

Cerebrovascular anatomical variants increase the challenge of endovascular clot retrieval in patients with MVOs. We report a case of acute anterior MVOs in a patient with DMCA. In 1962, Crompton first used the term accessory middle cerebral artery (AMCA) to describe the vessels that enter the Sylvian fissure with the MCA, including the AMCA and DMCA (8). Teal et al. proposed in 1973 that the term AMCA should be strictly limited to the anomalous arteries arising from the ACA, whereas branches arising from the ICA should be referred to as the DMCA (9). When one branch of the DMCA is occluded, the angiogram may confuse the operator and lead to a missed diagnosis because one branch of the DMCA is occluded while the other may show a normal middle cerebral artery, which requires careful screening. In the present case, there was no malignant middle cerebral artery occlusion syndrome, such as coma and cerebral edema. Considering the acute occlusion of the MCA, the DMCA can compensate for the blood supply to a certain extent and avoid the occurrence of large cerebral infarction (5). CTA was initially interpreted as no proximal MCA occlusion since the DMCA showed a distal M1 occlusion. However, a CTP scan showed a large ischemic penumbra, which was then suspected to be due to an emergent large vessel occlusion (LVO) not shown on CTA. This was then diagnosed by DSA as an occlusion of one of the two left MCAs proximal to the MCA, as well as segment A1 of the ACA. Therefore, in acute stroke patients with a high clinical suspicion of LVO, continued CTP is necessary even if the CTA is negative to avoid missing strokes in patients with DMCA or other similar vascular variants (5, 9).

Acute MVO frequently leads to adverse neurological outcomes, which may be partly due to the multisite thrombus burden precluding sufficient recanalization. How to perform MT in patients with MVO remains a technical challenge. Since occlusions occur at multiple sites at the same time, it is a challenge to decide how to prioritize the target vessels. A recent case series (treated with BGC, catheter aspiration, and stent retrievers) showed that the successful recanalization rates of MCA and ACA were 83.3 and 83.3%, respectively (4). Numerous clinical studies also have found that first-pass complete reperfusion of acute internal carotid artery occlusions, usually simultaneous involvement of MCA and ACA, is best achieved by combining a BGC with both DAC and stent retriever (10, 11).

To the best of our knowledge, the present case is the first reported case of an acute embolic occlusion of the multivessel with DMCA who underwent a thrombectomy where we used the combined BGC, SR, and DAC technique. We initially targeted the M1 segment for thrombus removal because the occluded segment was located proximal to the M1 segment. BGC was used to block the antegrade flow of ICA and SR combined with DAC for thrombus removal. Fortunately, the superior proximal M1 and A1 segments were removed simultaneously, and we found a thrombus on the stent and in the DAC. The BGC vacuum-assisted aspiration of DAC removed the thrombus from the A1 segment. The same procedure was repeated for the occlusion of the M2 segment of the DMCA and demonstrated complete recanalization with an mTICI score of 2b. To date, two cases of thrombectomy associated with DMCA have been reported. Koge et al. described vessel wall injury by a stent retriever device that caused failed recanalization in a case of ICA occlusion with concomitant MCA and DMCA occlusions (12). They reported that if MCA duplication was suspected from a lateral view microangiography, they might use a contact aspiration technique in the first-pass. There are several advantages to the use of combined techniques compared with single device techniques. First, SR allows distal capture of the thrombus while vacuum-assisted aspiration reaches the proximal side, allowing capture of the thrombus on both sides, thereby reducing the risk of thrombus fragmentation during thrombectomy (13). In addition, by capturing a portion of the SR through DAC, there is less contact between the SR and the artery wall, reducing radial and traction forces, and thus reducing the risk of vascular injury (13). The proximal block of the BGC further reduces the risk of thrombus escape to the distal ACA and MCA. At the same time, the vacuum state improves the aspiration of the DAC. Pressman et al. reported another case and showed that it was difficult to diagnose a proximal MCA stroke in a patient with DMCA, which missed the obstruction given the patency of the second MCA (5). Prompt diagnosis of this LVO is essential so that mechanical thrombectomy can be performed on the patient as soon as possible to restore perfusion to the penumbra before the temporary defect becomes permanent (5).

In conclusion, this case suggested that the DMCA may play a role in collateral flow around the main MCA occlusion and could confuse our decision-making. Owing to the anatomical characteristics of the DMCA, we should pay attention to the endovascular treatment strategy in case of concomitant ACA and DMCA occlusions.

## Take-away lessons

Identification of duplication MCA is challenging because, in these patients with acute ischemic stroke, CTA appears to show patency of all intracranial vessels and compensates to some extent for the blood supply, avoiding the development of a large cerebral infarction.

Combined balloon guide catheter with stent retriever and aspiration technique can increase the likelihood of first-pass recanalization in multivessel occlusions.

## Data availability statement

The raw data supporting the conclusions of this article will be made available by the authors, without undue reservation.

## Ethics statement

The studies involving human participants were reviewed and approved by Institutional Review Board of the Nanjing Medical University. The patients/participants provided their written informed consent to participate in this study.

## Author contributions

H-JH and Y-FW drafted the manuscript. QS, X-MW, and Y-FW added clinical data and revised the manuscript. All authors contributed to the article and approved the submitted version.
